# A Rice Immunophilin Gene, *OsFKBP16-3*, Confers Tolerance to Environmental Stress in *Arabidopsis* and Rice

**DOI:** 10.3390/ijms14035899

**Published:** 2013-03-13

**Authors:** Hyun Ji Park, Sang Sook Lee, Young Nim You, Dae Hwa Yoon, Beom-Gi Kim, Jun Cheul Ahn, Hye Sun Cho

**Affiliations:** 1 Green Bio Research Center, Korea Research Institute of Bioscience and Biotechnology, Daejeon 305-506, Korea; E-Mails: hotfehj@kribb.re.kr (H.J.P.); sslee@kribb.re.kr (S.S.L.); dudsla83@kribb.re.kr (Y.N.Y.); daehwa85@kribb.re.kr (D.H.Y.); 2 Department of Pharmacology, Medical Science, Seonam University, Namwon 590-170, Korea; 3 Division of Bio-Crops Development, National Academy of Agricultural Science, RDA, Suwon 441-707, Korea; E-Mail: bgkimpeace@korea.kr

**Keywords:** FK506 binding protein, FKBP16-3, thylakoid lumen, environmental stress tolerance

## Abstract

The putative thylakoid lumen immunophilin, FKBP16-3, has not yet been characterized, although this protein is known to be regulated by thioredoxin and possesses a well-conserved CxxxC motif in photosynthetic organisms. Here, we characterized rice *OsFKBP16-3* and examined the role of this gene in the regulation of abiotic stress in plants. FKBP16-3s are well conserved in eukaryotic photosynthetic organisms, including the presence of a unique disulfide-forming CxxxC motif in their *N*-terminal regions. *OsFKBP16-3* was mainly expressed in rice leaf tissues and was upregulated by various abiotic stresses, including salt, drought, high light, hydrogen peroxide, heat and methyl viologen. The chloroplast localization of OsFKBP16-3-GFP was confirmed through the transient expression of *OsFKBP16-3* in *Nicotiana benthamiana* leaves. Transgenic *Arabidopsis* and transgenic rice plants that constitutively expressed *OsFKBP16-3* exhibited increased tolerance to salinity, drought and oxidative stresses, but showed no change in growth or phenotype, compared with vector control plants, when grown under non-stressed conditions. This is the first report to demonstrate the potential role of FKBP16-3 in the environmental stress response, which may be regulated by a redox relay process in the thylakoid lumen, suggesting that artificial regulation of *FKBP16-3* expression is a candidate for stress-tolerant crop breeding.

## 1. Introduction

Immunophilins (IMMs) are receptors for the immunosuppressive drugs, cyclosporine A and FK506; the cyclophilin (CYP) and FK506 binding protein (FKBP) families can be distinguished based on their receptors [1]. IMMs contain a peptidyl prolyl isomerase (PPIase) domain, which catalyze a rate-limiting step in protein-folding by *cis/trans* isomerization of the proline imidic peptide bonds [2–4]. They are vital proteins in almost all organisms and are present in all major of subcellular organelles [5]. In plants, many IMMs are present in the chloroplast thylakoid lumen (TL), with 17 and 16 IMMs present in the thylakoid lumen in *Arabidopsis* and rice, respectively, as determined by analysis using TargetP, SignalP and TL proteomics [6–9]. The plant IMM family comprises the largest IMM family among all types of organisms studied.

TL compartments, which are surrounded by thylakoid membranes, play essential roles in oxygen evolution, adenosine triphosphate (ATP) formation and the maintenance of ion current balance during photosynthesis [10,11]. Recent proteomics studies reveal that TLs contain many novel protein families, such as IMMs (7 FKBPs and 3 CYPs), DegQ protease (3), PsbD (8), pentapeptide proteins (2) and ascorbate peroxidase (8). Except for oxygen-evolving complex proteins, plastocyanin, violaxanthin de-epoxidase, polyphenol oxidase and PsaN proteins, IMM is the largest protein family in the chloroplast lumen organelle [8,9]. Nevertheless, the exact biological and biochemical roles of most TL IMMs remain to be elucidated. Previously, we found that rice contains 29 genes encoding FKBPs and 27 genes encoding CYPs, among which 18 IMMs are localized to the chloroplast. Sixteen of these IMMs have signal peptides for lumen compartment localization, and only two IMMs may be localized to the stroma [7]. Therefore, TL IMMs may function as regulators of the photosynthetic process by assembling and maintaining the photosynthetic protein complex in the lumen. Plant IMMs have been classified in *Arabidopsis* and rice (and other monocots); the biological and physiological roles that some TL IMMs play in photosynthesis have been demonstrated. For example, AtFKBP13 has strong PPIase activity and plays a role in the accumulation of Reiske protein, a subunit of the cytochrome *b6f* complex, as well as redox regulation under high light conditions [12,13]. AtFKBP20-2 is involved in the accumulation of photosystem II (PSII) proteins [14], while AtCYP38 plays a vital role in PSII assembly and maintenance [15,16]. CYP38, whose crystal structure has recently been identified, interacts with the E-loop of chlorophyll protein 47 (CP47) [17]. AtCYP20-2, another IMM with strong PPIase activity that is located within the TL [18], has light-regulated expression [19] and is associated with PSII [20] and the NAD(H) dehydrogenase (NDH) complex in the TL [21]. Furthermore, rice *OsCYP20-2* is highly regulated by abiotic stress and functions in salinity and drought stress tolerance, as determined by the analysis of transgenic *Arabidopsis* and tobacco plants expressing this gene [22]. TaCYP20-2 regulates wheat stem development mediated by DELLA protein degradation [23]. AtFKBP16-2, another subunit of the NDH complex, plays an essential role in the stability of this complex [24]. Moreover, the roles of two TL IMMs (TaFKBP16-1 and TaFKBP16-3) were identified through yeast two-hybrid analysis, which led to the identification of proteins that specifically interact with these proteins (*i.e.*, PsaL, a subunit the PSI complex, and APO2/Tfh1 interacting with FKBP16-1 and FKBP16-3, respectively) [25]. TL IMMs may, therefore, function as fine-tuning regulators of photosynthetic apparatus in response to environmental changes and could be candidates for enhancing photosynthetic stress tolerance.

A common reaction to abiotic stress conditions, such as salinity, drought and high light, is the increased production of reactive oxygen species (ROS) within the plant cell compartment [26]. ROS, which are generated in response to various external and internal oxidative stimuli, cause oxidative cysteine thiol modifications, including the formation of disulfide bonds, in some proteins. Redox-active cysteine residues in these proteins are readily oxidized by ROS. Therefore, redox active cysteine residues play a key role in the redox regulation of the folding and stability of these proteins. The amino acid sequences and motifs of FKBP16-3 are relatively well conserved in photosynthetic plants and *Chlamydomonas*, especially the well-conserved redox active site CxxxC, which may form an intra-disulfide bond. Many reducing enzymes, including a family of thioredoxins [27], also facilitate reversible cysteine oxidation by promoting reducing conditions. FKBP16-3s are one of the targets of thioredoxin in *Arabidopsis*[28]. FKBP16-3s are also targets of glutathionylation in *Chlamydomonas reinhardtii*[29]. Thus, FKBP16-3s may function in redox regulation through thiol modification of their CxxxC motifs during photosynthesis and/or under abiotic stress conditions. Although FKBPs are involved in abiotic stress responses in plants [22] and FKBP of the TL is regulated by a redox system [13,14], no previous reports have focused on improving abiotic stress resistance via redox regulation of FKBP.

In this study, we characterized the function of rice FKBP16-3, an IMM that regulates gene expression in response to abiotic stress. Using a green fluorescent protein (GFP) reporter construct, we determined that OsFKBP16-3 is localized to chloroplasts in plant cells. *Arabidopsis* and rice plants that constitutively expressed *OsFKBP16-3* showed improved tolerance to salinity, drought and oxidative stress at various developmental stages. In addition, we confirmed that two cysteines (CxxxC) of recombinant OsFKBP16-3 exist in two forms, the oxidized and reduced form, based on the presence of dithiothreitol (DTT). These results suggest that FKBP16-3 plays a role in maintaining photosynthetic acclimation or the integrity of the photosystem apparatus under various environmental stresses. This is the first report that provides a functional characterization of FKBP16-3.

## 2. Results

### 2.1. FKBP16-3s Are Well Conserved in Photosynthetic Organisms

**Figure 1:**
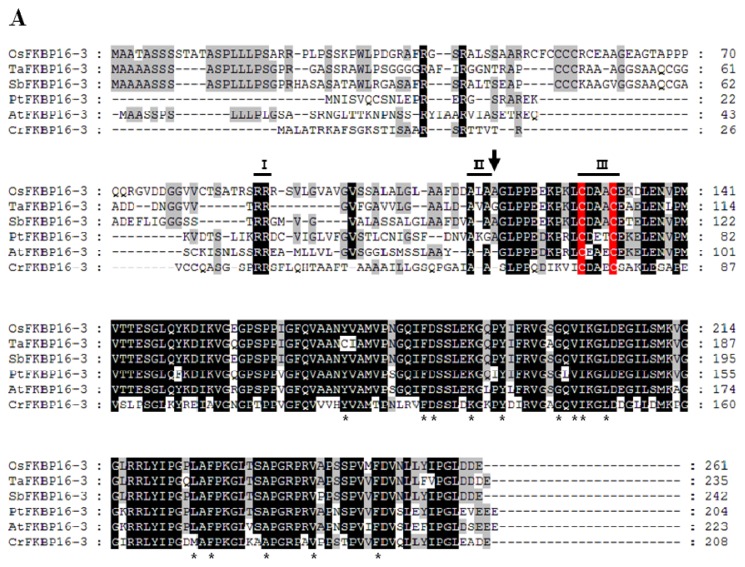

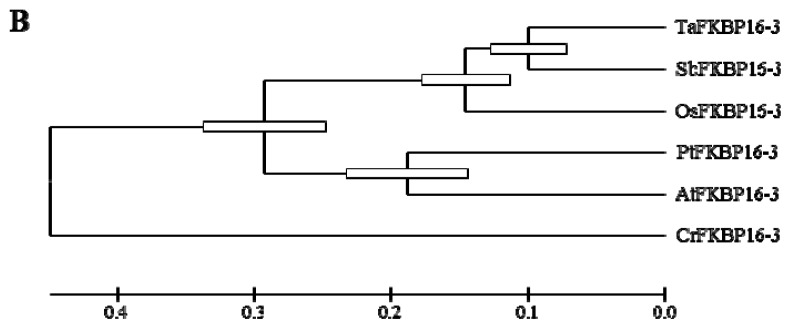
Multiple sequence alignment and phylogenetic relationship between OsFKBP16-3 and other FKBP16-3s. (**A**) Comparison of the deduced amino acid sequences of FKBP16-3s. The alignment was performed using the ClustalW2 and GeneDoc2.7 programs. Asterisks denote key residues for PPIase activity. The putative residues for chloroplast and thylakoid targeting are represented by I and II. The arrow indicates the predicated cleavage site between the transit peptide and the mature protein. The cysteine residues of FKBP16-3 are marked with a red background and III. The gradient shaded background indicates amino acid similarity (black > 90%, dark gray > 80%, light gray > 50%); (**B**) Phylogenetic distance between OsFKBP16-3 and other FKBP16-3s. The phylogenetic tree was constructed using the MEGA5 program. The accession numbers of FKBP16-3s are as follows: OsFKBP16-3 (*Oryza sativa*), NP_001062387.1; TaFKBP16-3 (*Triticum aestivum*), TA84905_4565; SbFKBP16-3 (*Sorghum bicolor*), XP_002445769.1; PtFKBP16-3 (*Populus trichocarpa*), XP_002328028.1; AtFKBP16-3 (*Arabidopsis thaliana*), At2g43560.1; CrFKBP16-3 (*Chlamydomonas reinhardtii*), XP_001692929.1.

OsFKBP16-3, which comprises 216 amino acids, contains a predicted bipartite chloroplast and TL target sequence at the *N*-terminus and a single FK506 binding domain (Figure 1A). We searched five putative FKBP 16-3 proteins by BLASTP using rice OsFKBP16-3 (LOC_08g42850) as the query, and we aligned the sequences to compare the conservation patterns of the amino acid residues using the GeneDoc program [30]. When we compared amino acid sequences, except the *N*-terminal signal peptides for chloroplast lumen targeting, four FKBP16-3 proteins displayed over 85% identity with OsFKBP16-3, including SbFKBP16-3 (97% identity), TaFKBP16-3 (91% identity), AtFKBP16-3 (86% identity) and PbFKBP16-3 (86% identity), while *Chlamydomonas* CrFKBP16-3 shares only 64% identity with OsFKBP16-3 at the amino acid level. OsFKBP16-3 is not highly similar to human FKBP12; OsFKBP16-3 has only seven of 14 key amino acid residues for FK506 binding/PPIase activity that are present in human FKBP12 [31–33] and lacks the PPIase activity detected in TaFKBP16-3 [25]. Other FKBP16-3s also showed partial similarity in terms of key amino acid residues for FK506 binding/PPIase activity. Nevertheless, sequence alignment indicated that FKBP16-3 proteins are highly conserved in photosynthetic organisms.

The TL FKBPs in *Arabidopsis* are encoded by nuclear genes and translocated to the chloroplast (specifically the thylakoid) through the action of cleaved *N*-terminal, bipartite signal peptides in precursor proteins [6]. The predicted thylakoid targets of the luminal FKBPs contain a twin arginine motif (RR), which functions in the “twin-arginine translocation” (TAT) pathway for thylakoid entry [34]. The thylakoid entry signal peptide RR, which functions in the TAT pathway and is a TL cutting motif, is well conserved in all FKBP16-3s; Ala-Xaa-Ala residues are relatively well conserved, although, in the *Sorghum bicolar* protein SbFKBP16-3, Ala-Xaa-Ala was changed to Val-Ala-Ala. Furthermore, AtFKBP16-3 is a TL protein, as determined by proteomic analysis of lumen-specific thylakoid fractions [9]. All FKBP16-3s have a unique CxxxC motif, which rapidly forms disulfide bonds under native conditions and is a major active site of thiolation by thioredoxin for redox regulation during cellular signal transduction [27]. This presence of the CxxxC motif in FKBP16-3 suggests that FKBP16-3 proteins would be able to play an essential role in TL that is related to the redox relay system, which helps plants overcome oxidative damage resulting from photosynthesis. Figure 1B shows the phylogenetic tree that was constructed (using MEGA software) to analyze the sequence alignment shown in Figure 1A. The amino acid sequences of the mature forms of FKBP16-3s are highly similar; phylogenetic tree analysis revealed that OsFKBP16-3s share high similarity in monocots (84% with Ta and 89% with Sb) and are less highly conserved in dicots (79% similarity with Pt and 78% with At).

### 2.2. Expression Analysis of *OsFKBP16-3*

**Figure 2:**
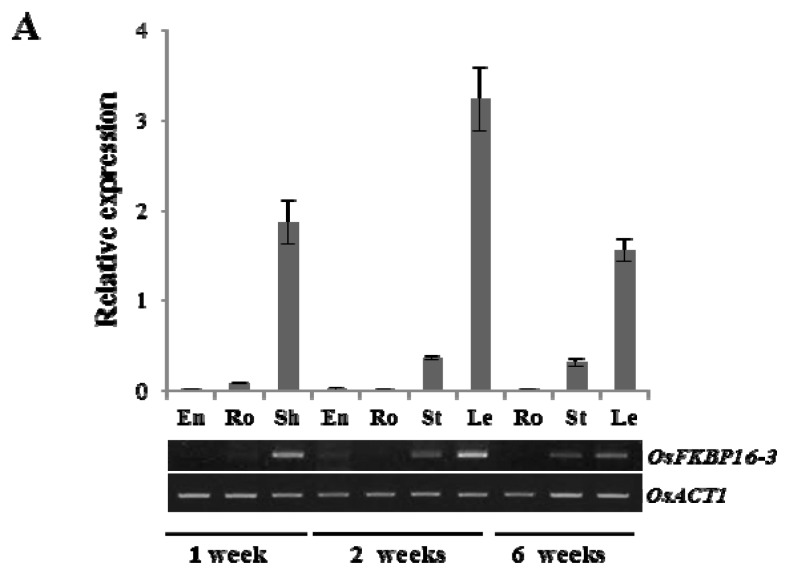

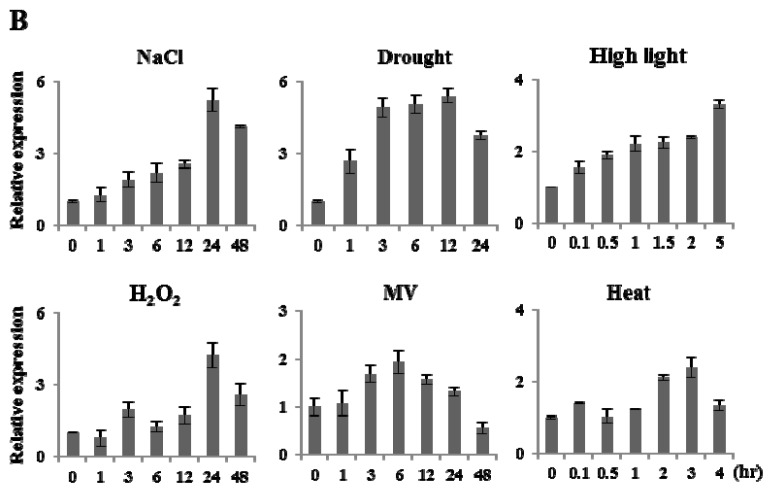
*OsFKBP16-3* gene expression levels. (**A**) Transcript levels of *OsFKBP16-3* gene in various tissues and developmental stages, as detected by semi-quantitative real-time polymerase chain reaction (RT-PCR) and real-time quantitative RT-PCR. cDNA templates from endosperm (En), root (Ro), sheath (Sh), stem (St) and leaf (Le) were used for amplification. Rice *Actin1* gene was used as a control; (**B**) Expression level of *OsFKBP16-*3 in rice seedlings under various stress conditions. RNAs were isolated from 10-day-old seedlings that were exposed to 200 mM NaCl, desiccation (for drought), 800 μmol photons m^2^ s^−1^ (for high light treatment), 10 mM H_2_O_2_, 10 μM methyl viologen (MV) or 42 °C heat at the indicated time points. Means and SD were calculated from three independent experiments.

To analyze the expression pattern of *OsFKBP16-3* during plant growth, cDNA templates were prepared from the mRNA of endosperms (En), roots (Ro), sheaths (Sh), stems (St) and leaves (Le) of one- and two-week-old seedlings and six-week-old plants. In addition, gene-specific primers were used for quantitative real-time polymerase chain reaction (qRT-PCR) and semi-quantitative RT-PCR (RT-PCR; Figure 2A). The expression of *OsFKBP16-*3 was higher in the photosynthetic leaf tissue than in other tissues, regardless of developmental stage. The highest level of *OsFKBP16-3* expression was in two-week-old leaves, while this gene was poorly expressed in the plastids and proplastids of nonphotosynthetic tissues, such as En and Ro. To further analyze the expression patterns of *OsFKBP16-3*, qRT-PCR analyses were conducted using cDNAs from seedlings subjected to various abiotic stresses, such as salt, drought, high light, hydrogen peroxide (H_2_O_2_), methyl viologen (MV) and heat. Most abiotic stresses produced a 2–5-fold increase in *OsFKBP16-3* expression. In particular, the expression of *OsFKBP16-3* was strongly increased under high salinity and drought stress conditions. Under salt stress (NaCl) conditions, *OsFKBP16-3* expression began to increase at 3 h, with a five-fold increase at 24 h, followed by a decrease after 48 h. Under drought stress conditions, *OsFKBP16-3* expression increased rapidly within 1 h of drought treatment, with an approximately 2.5-fold increase, with a four-fold increase at 12 h. As OsFKBP16-3 is a TL protein, we tested the level of *OsFKBP16-3* expression under high light stress. *OsFKBP16-3* expression rapidly responded to high light (within 10 min), with a three-fold increase in expression at 5 h. MV and H_2_O_2_, which stimulate ROS production, also affected the expression of *OsFKBP16-3* (from two- to four-fold). *OsFKBP16-3* expression increased slightly in response to heat stress, with a two-fold increase within 2 h of treatment (Figure 2B). These results indicate that *OsFKBP16-3* is expressed in all photosynthetic green tissues, with little expression in nonphotosynthetic tissues. Moreover, *OsFKBP16-3* expression was highly regulated by various environmental stresses, which suggests that OsFKBP16-3 is involved in TL stress defense mechanisms or photosynthetic acclimation to environmental stresses.

### 2.3. OsFKBP16-3 Is Localized to the Chloroplasts in Plant Cells

**Figure 3:**
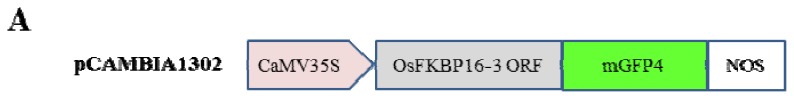

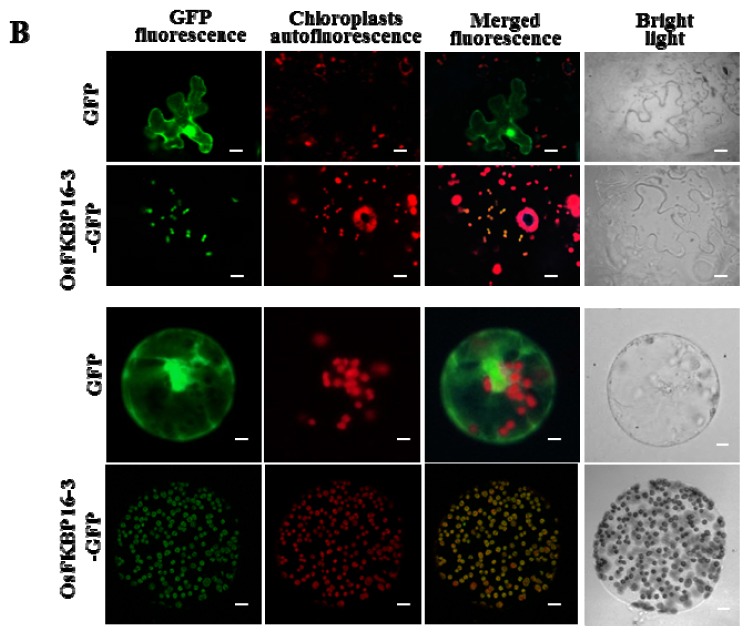
Subcellular localization of OsFKBP16-3 by transient expression of the green fluorescent protein (GFP) fused fluorescent proteins. (**A**) Structure of pCAMBIA 1302 binary constitutive expression vector harboring *OsFKBP16-3*; (**B**) Microscopic images of GFP, chloroplast autofluorescence and merged fluorescence from epidermal cells and protoplasts of *Nicotiana benthamiana* infected with *Agrobacterium* GV3101 harboring the GFP constructs. GFP alone (negative control) and GFP fused to OsFKBP16-3. The photographs were taken in the blue channel, red channel or combination. Bar = 10 μM.

To determine the localization of OsFKBP16-3 protein in the plant cell, we fused the open reading frame of OsFKBP16-3 to the sequence encoding the *N*-terminus of GFP protein in the pCAMBIA 1302 vector (Figure 3A) and used this construct to transiently transfect *N. benthamiana* leaves. The expression of OsFKBP16-3-GFP was confirmed using the GFP antibody (data not shown). Although the exact TL localization could not be verified, Figure 3B shows that OsFKBP16-3-GFP protein was expressed in *N. benthamiana* chloroplasts. These results are consistent with the localization of Arabidopsis FKBP16-3 that was predicted using SignalP, TargetP and Predotar software, as well as TL proteomic analysis [8,9]. The autofluorescence of the chloroplasts was clearly merged with the OsFKBP16-3-GFP signal, as observed by fluorescence microscopy. Therefore, OsFKBP16-3 is a chloroplast protein.

### 2.4. Ectopic Expression of *OsFKBP16-3* Increased Tolerance to Salinity in Arabidopsis

**Figure 4:**
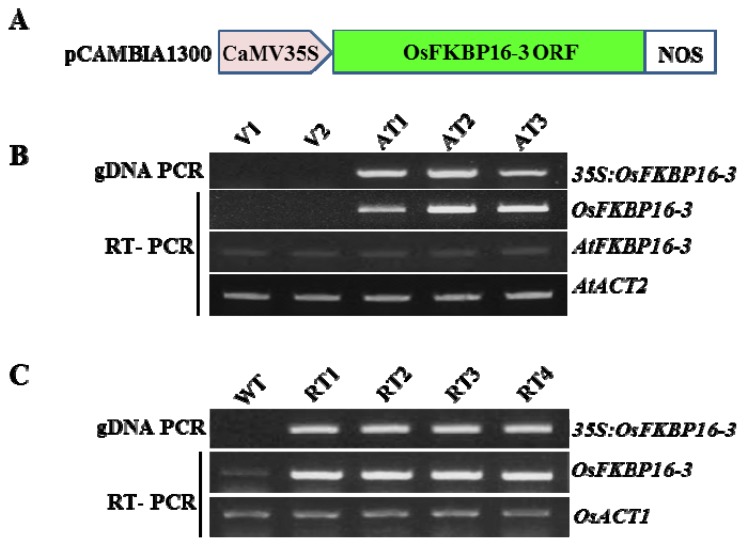
Constitutive expression of *OsFKBP16-3* in *Arabidopsis* and rice. (**A**) Structure of the pCAMBIA1300 binary vector harboring *OsFKBP16-3* under the control of the 35S promoter; (**B**,**C**) Constitutive expression of *OsFKBP16-3* in *Arabidopsis* and rice. RT-PCR was performed with vector control and *OsFKBP16-3*-overexpressing transgenic plants. *OsACT1* was used as a control for mRNA normalization. V: pCAMBIA vector control transgenic plants; AT: *OsFKBP16-3*-expressing transgenic *Arabidopsis* plants; WT: wild-type rice, RT: *OsFKBP16-3-*overexpressing transgenic rice plants.

We generated *OsFKBP16-3*-expressing transgenic Arabidopsis using the pCAMBIA 1300 vector, which contains *OsFKBP16-3* under the control of the constitutive 35S promoter, as shown in Figure 4A. The ectopic expression of *OsFKBP16-3* was confirmed by confirming *35S:OsFKBP16-3* DNA insertion into the genome and *OsFKBP16-3* expression was analyzed, as shown in Figure 4B. Three independent, homozygous transgenic Arabidopsis lines showed ectopic expression of *OsFKBP16-3*. The AT2 and AT3 transgenic lines had higher levels of *OsFKBP16-3* expression than AT1 or either of the two vector control lines, with V1 and V2 showing no *OsFKBP16-3* expression. The native *AtFKBP16-3* transcript was expressed at the same level in all transgenic Arabidopsis lines. *OsFKBP16-3* was highly overexpressed in young transgenic rice plants compared with the wild-type (Figure 4C).

**Figure 5:**
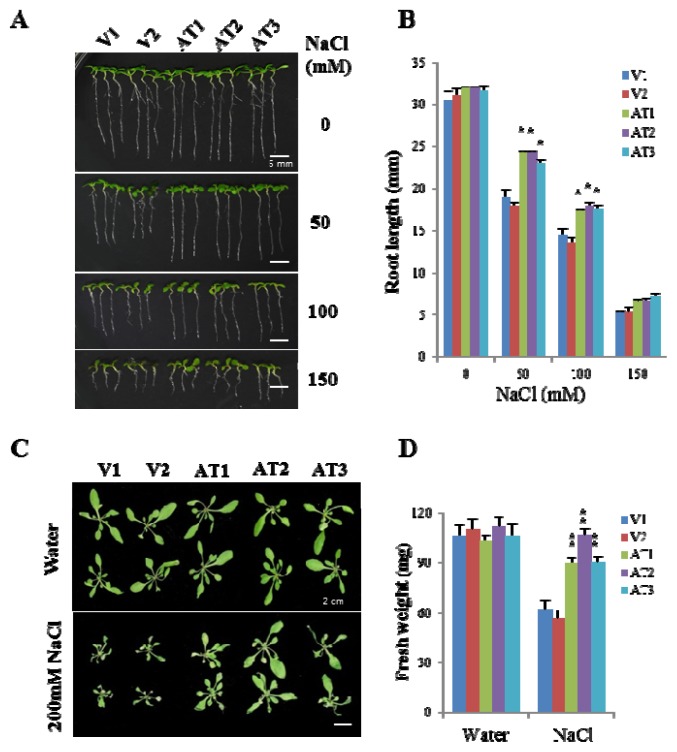
Performance of *OsFKBP16-3*-overexpressing transgenic *Arabidopsis* under salt stress. (**A**) Comparison of *Arabidopsis* seedlings under salt stress. Seeds of vector control and *OsFKBP16-3*-expressing transgenic *Arabidopsis* lines were exposed to Murashige and Skoog (MS) medium containing 0, 50, 150 or 200 mM salt for 10 days; (**B**) Root lengths of transgenic and vector control seedlings grown under salt stress; (**C**) Comparison of two-week-old *Arabidopsis* plants grown in soil under salt stress for seven days; (**D**) Fresh weights of transgenic and vector control plants. V: pCAMBIA vector control transgenic plants, AT: *OsFKBP16-3*-expressing transgenic *Arabidopsis*. Error bars represent SD. Asterisks indicate significant differences from the vector control using *t*-test *p*-value. (*t-test, with *p* < 0.01, **t-test, with *p* < 0.001)

First, to examine whether the constitutive expression of *OsFKBP16-3* increased abiotic stress tolerance in transgenic *Arabidopsis* plants, we exposed five-day-old vector control and *OsFKBP16-3* transgenic *Arabidopsis* seedlings to Murashige and Skoog (MS) medium containing 0, 50, 150 or 200 mM NaCl for 10 days. As shown in Figure 5A, the vector control lines V1 and V2 exhibited retarded plant growth in proportion to the salt concentration, whereas the *OsFKBP16-3 Arabidopsis* lines AT1, AT2 and AT3 exhibited more tolerance to NaCl than the vector controls, depending on the NaCl concentration. We measured the root lengths of transgenic seedlings of vector controls and *OsFKBP16-3* seedlings under salt stress (Figure 5B). At a NaCl concentration of 50 or 100 mM, all of the *OsFKBP16-3* transgenic lines showed obvious differences in root length compared with vector controls, with *p* < 0.01 or 0.001, but the root lengths were similar under normal conditions. To test the NaCl tolerance of *Arabidopsis* plants at different developmental stages, two-week-old soil-grown plants were treated with NaCl. Salinity treatment (200 mM NaCl) dramatically affected the growth of vector control plants, which exhibited reduced leaf length and width, but less damage was seen in *OsFKBP16-3* transgenic plants (Figure 5C). Although chlorosis and growth retardation were seen in both vector control and *OsFKBP16-3*-expressing transgenic plants after four days of salt treatment compared with water-treated plants, *OsFKBP16-3* transgenic plants were less affected by salt, with more greening and growth than vector control plants. The three transgenic lines had significantly higher fresh weights (maximum of two-fold higher) under NaCl conditions compared with the vector control plants, while no significant difference in fresh weight was observed between vector control and transgenic plants under normal watering conditions (Figure 5D). Therefore, OsFKBP16-3 probably plays an important role in environmental or NaCl stress, regardless of developmental stage.

### 2.5. Ectopic Expression of *OsFKBP16-3* Showed Increased Tolerance to Drought Stress in Arabidopsis

**Figure 6:**
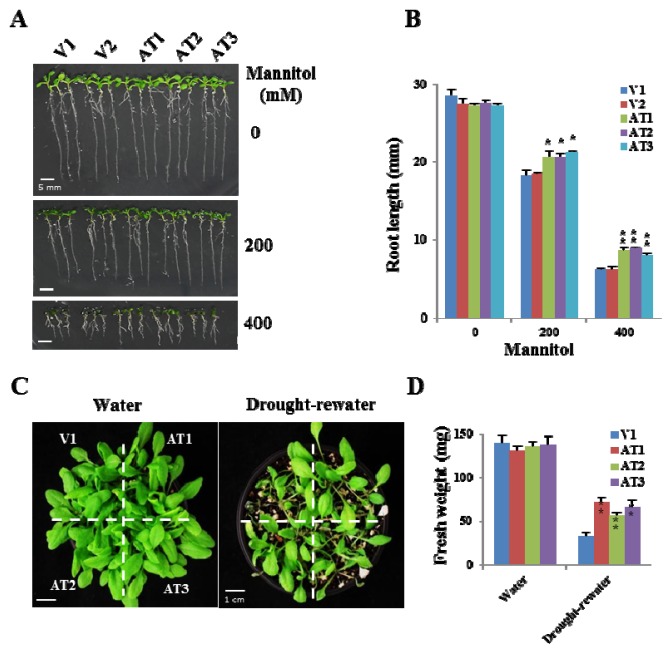
Performance of *OsFKBP16-3* transgenic *Arabidopsis* under drought stress. (**A**) Comparison of *Arabidopsis* seedlings under mannitol stress. Four-day-old seedlings of vector control and *OsFKBP16-3*-expressing transgenic *Arabidopsis* lines were exposed to MS medium containing 0, 200 or 400 mM mannitol for 10 days; (**B**) Root lengths of transgenic and vector control seedlings under mannitol stress; (**C**) Comparison of *Arabidopsis* plants grown in soil under desiccation stress. Two-week-old transgenic *Arabidopsis* plants were exposed to drought stress by withholding watering for eight days, followed by re-watering. The photographs were taken immediately after one day of re-watering; (**D**) Measurement of fresh weights of transgenic and vector control plants after drought stress treatment. V: pCAMBIA vector control transgenic plants; AT: ectopic expression of *OsFKBP16-3* transgenic *Arabidopsis*. Error bars represent SD; asterisks indicate significant differences from the vector control using *t*-test *p*-value. (*t-test, with *p* < 0.1, **t-test, with *p* < 0.05).

Since the expression of *OsFKBP16-3* was also increased in response to drought stress, we conducted experiments to test the drought stress tolerance of *OsFKBP16-3 Arabidopsis* plants. We transferred four-day-old seedlings to Murashige and Skoog (MS) medium containing 0, 200 or 400 mM mannitol and grew the seedlings for 10 days. Although the root lengths of the seedlings were dramatically reduced in proportion to mannitol concentration, the *OsFKBP16-3* lines showed better root growth than the vector controls (Figure 6A,B). The root lengths of *OsFKBP16-3* plants treated with 400 mM mannitol were over 1.5-fold those of vector control seedlings for all *OsFKBP16-3* lines. In addition, we conducted a desiccation assay using young soil-grown transgenic plants. Two-week-old *Arabidopsis* plants grown in soil were subjected to desiccation by withholding watering for eight days, followed by one day of re-watering (or no re-watering), to analyze desiccation tolerance. As shown in Figure 6C, there was no difference between vector control and *OsFKBP16-3* lines under normal watering conditions. However, the *OsFKBP16-3* lines exhibited notable drought tolerance compared with the vector control lines. Under desiccation conditions, most of vector control plants died, whereas the *OsFKBP16-3* lines were markedly less affected by desiccation, with less withering and a higher biomass than the controls. To quantify the increased drought tolerance of *OsFKBP16-3* lines, the aerial parts of transgenic plants were measured under normal watering and desiccation conditions. There was no difference in fresh weight between the control and *OsFKBP16-3* plants under normal watering conditions, but the aerial parts of the *OsFKBP16-3* plants weighed significantly (more than two-fold) more than those of the control plants under desiccation conditions (Figure 6D). From the desiccation assay results, we conclude that OsFKBP16-3 may be involved in a mechanism for overcoming drought stress within the TL.

### 2.6. Overexpression of *OsFKBP16-3* Improved Tolerance to Environmental Stress in Rice

**Figure 7:**
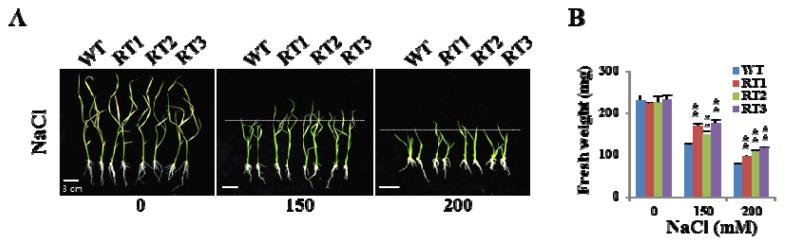

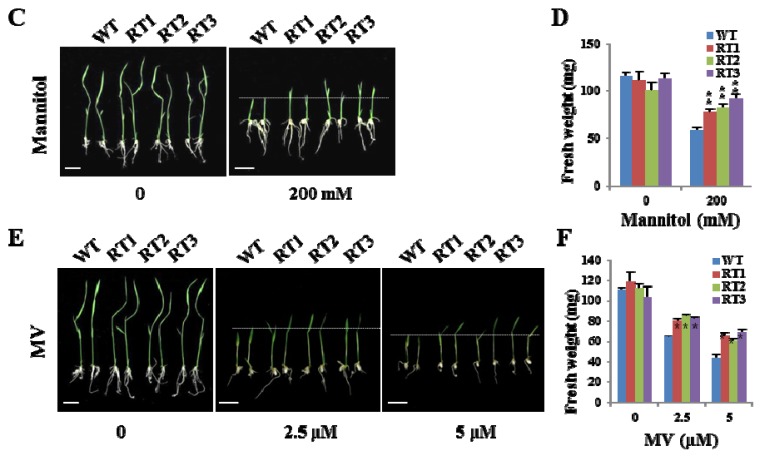
Performance of *OsFKBP16-3*-overexpressing transgenic rice under environmental stress. (**A**,**C**,**E**) Comparison of rice seedlings under NaCl, mannitol and methyl viologen (MV) stress. Transgenic plants were selected for three days on MS medium containing hygromycin (50 mg/L) and grown on fresh MS medium containing 0, 150 or 200 mM NaCl for 10 days (**A**); 200 mM mannitol for seven days (**C**) or 2.5 or 5 μM MV for seven days (**E**); (**B**,**D**,**F**) Fresh weights of transgenic and wild-type seedlings under environmental stress. WT: wild-type rice (Dongjin ecotype); RT: *OsFKBP16-3*-overexpressing transgenic rice. Error bars represent SD; asterisks indicate significant differences from the vector control using *t*-test *p*-value. (*t-test, with *p* < 0.1, **t-test, with *p* < 0.05).

To investigate the roles of OsFKBP16-3 in plants, we produced transgenic rice plants that constitutively expressed *OsFKBP16-3*, as shown in Figure 4C. To examine whether the overexpression of *OsFKBP16-3* confers increased tolerance to environmental stresses, we performed salinity, mannitol and MV tolerance assays with transgenic rice plants that were similar to those performed with *Arabidopsis*. We selected three overexpressing lines (RT1, RT2 and RT3) and the wild-type (var. Dongjin) for the tolerance assay. Transgenic rice plants were selected on MS medium containing hygromycin (50 mg/L) for three days, transferred to fresh MS medium containing 0, 150 or 200 mM NaCl and grown for 10 days. In addition, some of the selected plants were transferred to MS medium containing 200 mM mannitol, 2.5 μM MV or 5 μM MV and grown for seven days. Under normal conditions, there was no phenotypic difference between wild-type and *OsFKBP16-3*-overexpressing lines, whereas the overexpressing lines showed better growth than wild-type under environmental stress conditions (Figure 7A,C,E), although the differences between the lines were not remarkable. To quantify the phenotypic differences under stress conditions, we measured the fresh weights, shoot lengths and root lengths of the plants. There was a meaningful difference in fresh weight in all of three lines (Figure 7B,D,F), as well as significant differences in shoot length under stress conditions (data not shown). However, we did not detect clear differences in root length between the lines under stress conditions. These results demonstrate that the artificial upregulation of *FKBP16-3* may be a practical means of improving stress tolerance in crops.

### 2.7. Redox State of Recombinant OsFKBP16-3 Protein

**Figure 8:**
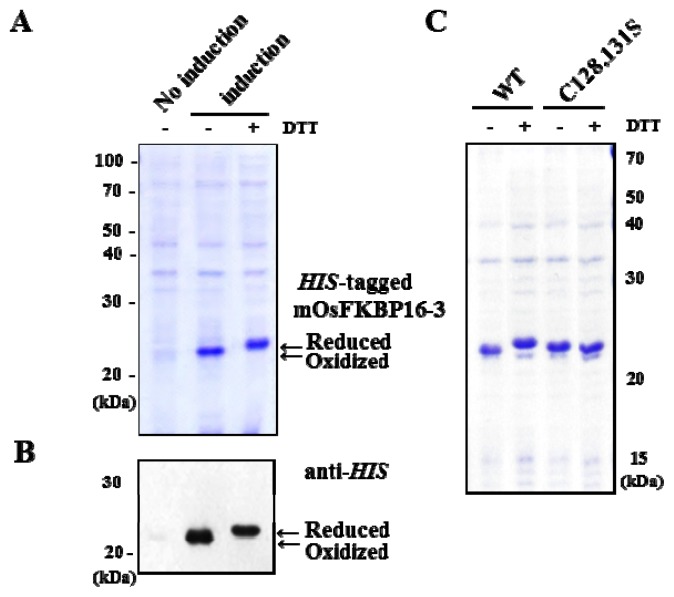
Expression and redox state of recombinant *his*-tagged mature form OsFKBP16-3 protein (mOsFKBP16-3) in *E. coli*. (**A**) Recombinant mOsFKBP16-3 protein expression was induced with isopropyl β-D-1-thiogalactopyranoside (IPTG) in *E. coli*. Samples were separated by 12% sodium dodecyl sulfate polyacrylamide gel electrophoresis (SDS-PAGE) under non-reducing conditions and stained with Coomassie blue. The reduction of *his*-tagged mOsFKBP16-3 protein was accomplished by treatment with 100 mM DTT; (**B**) Immunoblot of expressed OsFKBP16-3 protein probed with the monoclonal *his* antibody; (**C**) Two cysteine-to-serine mutation was carried out in mOsFKBP16-3 proteins. The mutant protein was expressed and treated with DTT as wild-type mOsFKBP16-3. WT: wild-type mOsFKBP 16-3 protein; C128,131S: cysteine to serine mutant protein of mOsFKBP16-3.

To confirm that OsFKBP16-3, which contains one pair of cysteine residues (the CxxxC motif), is regulated by redox, we expressed the mature form of OsFKBP16-3 (mOsFKBP16-3), without the predicted signal peptides, as *N*-terminal His-tagged recombinant protein in *E. coli* using the pET-28a (+) vector. Under non-reducing condition, recombinant mOsFKBP16-3 was induced by isopropyl β-d-1-thiogalactopyranoside (IPTG) and expressed as a soluble protein approximately 22 kDa in size (Figure 8A, lane 2), which roughly corresponds to the molecular weight of mOsFKBP16-3 protein (18.1 kD) combined with the 6× *his*-tag (0.8 kDa) and the translated vector (3.1 kDa). By contrast, *E. coli* cells that were not induced with IPTG expressed little protein of this molecular weight (Figure 8A, lane 1). Furthermore, *his*-tagged mOsFKBP16-3 recombinant protein exhibited immunoreactivity with the *his* antibody and produced a band consistent with the molecular weight of recombinant mOsFKBP16-3 (Figure 8B). DTT is frequently used to reduce the disulfide bonds of proteins and peptides. DTT prevents intra-molecular and inter-molecular disulfide bond formation between the cysteine residues of proteins. The reduction of *his*-tagged OsFKBP16-3 protein was accomplished by treatment with 100 mM DTT. OsFKBP16-3 protein treated with DTT showed slower mobility (reduced form) than untreated OsFKBP16-3 protein, as determined by 12% sodium dodecyl sulfate polyacrylamide gel electrophoresis (SDS-PAGE) under non-reducing conditions. However, the cysteine to serine mutants (C128,131S) of mOsFKBP16-3 were found to have the same mobility regardless with DTT treatment when expressed and run under the same conditions as wild-type mOsFKBP16-3 (Figure 8C). These results indicate that the two cysteine residues of CxxxC motif in OsFKBP16-3 protein may be involved in a redox-regulated system through the formation of an intra-molecular disulfide bridge.

## 3. Discussion

In a previous study, we found that significant numbers of rice *IMM* genes are upregulated in response to water stress. Among these genes, the expression levels of some *TL IMMs* are upregulated in response to water stress, such as salinity and desiccation stress, in addition to high light stress [7]. Some *TL IMMs* are more responsive to specific stresses than others. For example, *OsCYP20-2* and *OsCYP28* are upregulated by both salt and drought stress, but the expression of *OsFKBP16-1* and *OsFKBP16-3* is increased by salinity and desiccation, respectively [7] (Figure 2B). However, almost all *TL IMMs* are rapidly upregulated by high light levels [6]. A number of TL IMMs are involved in the assembly and/or maintenance of the photosynthetic apparatus [14,16,20,35], but the roles of IMMs in TL remain to be elucidated on the understanding of a role between photosynthesis and stress conditions.

ROS, which are generated by various environmental and developmental stimuli, can act as signaling molecules during stress adaptation, plant development and programmed cell death [26]. A slight alteration in the homeostatic set point of intracellular ROS level signals the cell to modulate its metabolism, gene expression and posttranslational modification of proteins [36–38], indicating that ROS are important for regulating normal cellular functions. In particular, when ROS levels exceed the cellular antioxidant capacity, cellular homeostasis is altered, which results in oxidative injury. Although the details of how ROS affects various cellular responses remain unclear, accumulating evidence suggests that ROS function as part of a signaling network in which the reversible oxidation-reduction of protein thiols serves as a switch for redox control circuits to regulate antioxidant defenses and specific cell signaling pathways.

The light-driven mechanism of photosynthesis also involves redox regulation in response to various environmental changes, which directly or indirectly affect the electron transport capability of the photosynthetic apparatus [39]. In chloroplasts, ROS are generated by environmental stresses, such as high light, low temperature and drought. Simultaneously, the oxidative damage caused by ROS is lessened by the scavenging activity of both non-enzymatic and enzymatic antioxidants [39,40]. Ferredoxin, FTR (ferredoxin:thioredoxin reductase) and thioredoxins are important disulfide-reducing regulatory proteins in chloroplasts that act as redox relays to target enzyme redox signals. Numerous chloroplast enzymes are targets for redox regulation by the ferredoxin/Trx system (such as FBPase, RuBisCO activase, ATP synthase and others) due to the presence of two cysteines for their disulfide bridges [41].

FKBP16-3 was identified as a thioredoxin-regulated protein through proteomic analysis of *Arabidopsis*[26]. In addition, two partner proteins of TaFKBP16-3, APO2 (accumulation of photosystem one 2) and Thf1 (thylakoid formation 1), were recently verified by yeast two-hybrid analysis [25]. APO2 contains well-conserved (4Fe-4S) cluster-binding motifs, existing in two CxxC sites, and it appears to play an important role in the stabilization of PsaA and PsaB [42–44]. *Thf1* expression is induced by light and controls an important step required for the normal organization of mature thylakoid membrane stacks [45]. Therefore, FKBP16-3 may acts as a redox relay in the assembly or maintenance of PSI and/or thylakoid membrane formation, although the exact biochemical function of FKBP16-3 remains to be elucidated. Although FKBP16-3 may play an important role in photosynthesis, the gene encoding this protein has not previously been characterized. Here, we characterized rice *OsFKBP16-3* by performing molecular and genetic analysis of this gene in *Arabidopsis* and rice. OsFKBP16-3 is a chloroplast lumen IMM that is well conserved in photosynthetic organisms, but this protein lacks essential amino acid residues for PPIase/FK506-binding activity (Figure 1A,B). *OsFKBP16-3* was expressed in almost all photosynthetic tissues, but was not expressed in roots, which do not contain chloroplasts and do not perform photosynthesis (Figure 2A). In addition, the expression of *OsFKBP16-3* was upregulated in response to various ROS-elevating conditions, such as high salinity, drought, high light, heat and treatment with the ROS-producing chemicals, H_2_O_2_ and MV (Figure 2B). We verified that OsFKBP16-3 is localized to chloroplasts by examining the expression of a GFP fusion protein in transiently transfected *Nicotiana benthamiana* leaves (Figure 3A,B), which is in agreement with previous genomic and proteomic studies demonstrating that FKBP16-3 exists in the TL in *Arabidopsis* and spinach [8,9]. Although we did not aim to discover the exact biochemical role of OsFKBP16-3 in photosynthesis, we examined whether the constitutive expression of *OsFKBP16-3* increases abiotic stress tolerance in *Arabidopsis* and rice plants. Ectopic expression and overexpression of *OsFKBP16-3* in *Arabidopsis* and rice (Figure 4B,C) provided oxidative, salinity and drought stress tolerance (Figures 5–7), as expected. The redox active sites in OsFKBP16-3 (*i.e.*, the CxxxC motif) were present in two forms (reduced and oxidized), depending on the presence and absence of DTT (Figure 8). These results led to the speculation that FKBP16-3 is an essential TL IMM for regulating redox balance during PSI assembly or thylakoid membrane biogenesis. By performing biochemical characterization of OsFKBP16-3 to help elucidate the exact physiological role of this protein in redox regulation in chloroplasts in conjunction with partner proteins, we hope to discover another unique redox mechanism of TL IMMs. Future studies will focus on the relationship between proteins that interact with OsFKBP16-3 and the redox states of OsFKBP16-3.

## 4. Experimental Section

### 4.1. Plant Growth Conditions and Treatments

All plant materials used in this study were in the Dongjin ecotype background of *Oryza sativa* or the Columbia-0 ecotype (Col-0) background of *Arabidopsis thaliana. Arabidopsis* seeds were sterilized and placed into plates containing MS medium with 0.3% agar and 1% sucrose. After stratification in the dark at 4 °C for 2 days, the plates were transferred to a growth chamber under white light (70 μmol m^−2^ s^−1^). Rice seeds were placed into Yoshida nutrient solution. The plants were grown at 28 °C for 1–2 weeks under a 12 h light/12 h dark cycle and subjected to stress treatments as described previously [7]. The seedlings were exposed to 200 mM NaCl (for salt treatment), desiccation (for drought treatment), 800 μmol photons m^−2^ s^−1^ (for high light treatment), 10 mM H_2_O_2_, 10 μM MV or 42 °C (for heat treatment) and harvested at various time points. Three experiments were performed per treatment, with at least three replicated measurements for each parameter assayed. For transient expression of OsFKBP16-3-GFP, *Nicotiana benthamiana* seeds were germinated and grown under a 16 h light/8 h dark cycle at 25 °C for 3 weeks prior to transfection.

### 4.2. Gene Expression Analysis

Using RNAiso Plus (TaKaRa Bio Inc., Dalian, China), RNA was extracted from plants grown under normal or stress conditions, and cDNA synthesis was performed, as previously described [7], with some modifications. For tissue-specific analysis, leaf, stem or root tissues were harvested from three randomly selected plants and pooled together to produce one sample. After RNase-free DNase I (RQ1 RNase-Free DNase; Promega, Madison, WI, USA) treatment, 3 μg of RNA was used for first-strand cDNA synthesis (RevertAid First-strand cDNA Synthesis Kit; Fermentas, Burlington, Canada). Real-time qRT-PCR reactions were performed in a 7500 Fast Real-Time PCR instrument (Applied Biosystems, Foster City, CA, USA) using SYBR Premix Ex Taq (TaKaRa Bio Inc., Dalian, China), according to the manufacturer’s instructions. The PCR reactions were performed using the primers listed in Supplementary Table S1. Gene-specific primers were designed, and all reactions were performed in triplicate. Relative expression levels represent the values relative to that of the corresponding control sample at the indicated time point, after normalization to actin transcript levels.

### 4.3. Localization of *OsFKBP16-3*

The *35S:OsFKBP16-3-GFP* gene in the pCAMBIA1302 binary vector was transiently expressed in *Nicotiana benthamiana* leaves infiltrated with *Agrobacterium* strain GV3101 carrying the appropriate binary plasmid. After 3 days of plant growth in a greenhouse at 26 °C under a 16 h light/8 h dark cycle, the infiltrated leaves were examined by fluorescence or confocal microscopy [22].

### 4.4. Gene Constructs and Plant Transformation

The vector for the constitutive expression of *OsFKBP16-3* was constructed by directionally inserting the full cDNA sequence of *OsFKBP16-3* into the pCAMBIA1300 vector under the control of the 35S promoter. The construct was transformed into *japonica* rice cv. Dongjin by *Agrobacterium*-mediated transformation [46] to generate transgenic rice plants. The same constructs were also introduced into *Arabidopsis* plants using the floral dip method [47]. Transgenic seeds (T_2_ and T_3_ generation, rice and *Arabidopsis*, respectively) were surface-sterilized and germinated on MS agar plates containing 20 mg l-1 hygromycin. Seven-day-old seedlings were transferred to soil and grown under a 16 h light/8 h dark cycle.

### 4.5. Molecular Analysis of Transgenic Plants

Genomic DNA isolated from the apical leaves of transgenic and control plants (grown in a greenhouse or in the field) was analyzed by PCR to detect the inserted *35S:OsFKBP16-3* by amplifying the inserted fragments with the forward primer of *OsFKBP16-3* and the reverse primer of the NOS-terminator following the protocol in Supplementary Table S1. RT-PCR was carried out with gene-specific *OsFKBP16-3* primers to amplify the transcript. DNA and RNA extracted from untransformed or vector control plants were used as negative controls and pCAMBIA-*OsFKBP16-3* DNA was used as a positive control. The PCR conditions were as follows: 94 °C for 3 min followed by 25 cycles of 94 °C for 45 s, 55 °C for 45 s and 72 °C for 1 min, followed by 72 °C for 7 min. The amplified PCR fragments were separated on a 0.01 g/mL agarose gel and observed under ultraviolet light.

### 4.6. Stress Treatment of Transgenic Plants

After *Arabidopsis* or rice seedlings were selected on medium containing hygromycin for 3–4 days, transgenic seedlings were transferred to MS medium supplemented with 0, 50, 100 or 150 mM NaCl, 0, 200 or 400 mM mannitol or 2.5 or 5 μM MV and grown for 7–10 days. For stress treatment of soil-grown plants, *Arabidopsis* or rice seedlings selected on medium containing hygromycin were transferred to soil and grown for 14 days. Drought stress studies were carried out by withholding water for 8 days. For salinity stress, soil-grown plants at the same stage were irrigated with 200 mM NaCl solution every other day for up to 7 days.

### 4.7. Protein Expression and Reduction of *OsFKBP16-3* in *E. coli*

The mature form *OsFKBP16-3* (*mOsFKBP16-3*) was cloned into pET28a (Novagen, Damstadt, Germany) and sequenced. The *OsFKBP16-3* construct was transformed into BL21 DE(3) pLys S *E. coli* for the expression of *his*-tagged mOsFKBP16-3 protein. Recombinant protein was overexpressed, following the manufacturer’s instructions. Successful protein expression was verified using SDS-PAGE. Detection of recombinant *his*-OsFKBP16-3 protein in *E. coli* was performed using universal Western analysis with the *his* antibody as a probe. Cysteine-to-serine mutagenesis was carried out by an overlap extension approach. Primers were designed to substitute two cysteines with serines. Two double serine mutants were constructed and expressed, as above.

## 5. Conclusions

The chloroplast (possibly TL) location of OsFKBP16-3 is confirmed. OsFKBP16-3 is not conserved in essential amino acids needed for PPIase activity, and the activity is not clear. However, as most others of the TL FKBP family, it seems clear that FKBP16-3 plays a role in the photosynthetic acclimation or the adaptation of plants against stress or environmental changes. The biochemical mechanism of the role is predicted to be regulated through a redox relay of two adjacent cysteines. Considering that it is needed to overcome the limitations of plant cultivation and to counteract to climate changes, such as global warming, the artificial and fine regulation on *FKBP16-3* is expected to play a role in the development of less sensitive crops on environmental stresses.

## Supplementary Information
