# Supplementary material for: A Rice Immunophilin Gene, OsFKBP16-3, Confers Tolerance to Environmental Stress in Arabidopsis and Rice
Source: Int J Mol Sci. 2013 Mar 13;14(3):5899–919. doi: 10.3390/ijms14035899 (PMC3634459; doi:10.3390/ijms14035899)
Supplement: Supplementary file 1 [file ijms-14-05899-s001.pdf]

Supplementary Information

Table S1. Gene specific primers used for PCR and cloning.

Gene Name	Primer Sequences (F, forward; R, reverse[5'-3'])	Applications
OsFKBP16-3	F: atggctgcaaccgcttcgtcctcctcc<br>R: tcactcatcatcaagactggtatgtac	RT-PCR, qPCR
OsACT1	F: catgctatccctcgtctcgacct<br>R: cgcacttcatgatggagttgtat	RT-PCR, qPCR
tNOS	R: ataatcatcgcaagaccggcaac	Genomic DNA PCR
OsFKBP16-3	F: caaagatctcatggctgcaaccgcttcg<br>R: cggagatctccctcatcatcaagacctgg	pCAMBIA1302 cloning
OsFKBP16-3	F: ggattcatggctgcaaccgcttcgtcctcctcc<br>R: ggatcctcactcatcatcaagactggtatgtac	pCAMBIA1300 cloning
AtFKBP16-3	F: gcagctggtttacctcctccag<br>R:cgtcaaatatcaccgggctg	RT-PCR
AtACT2	F: ggaaggatctgtacggtaac<br>R: tgtgaacgattcctggacct	RT-PCR
OsFKBP16-3	F: atccatatggtgggggtatcgtcggcgctg<br>R: aatctcgagctcatcatcaagacctggtatgtac	pET28a cloning

© 2013 by the authors; licensee MDPI, Basel, Switzerland. This article is an open access article distributed under the terms and conditions of the Creative Commons Attribution license (http://creativecommons.org/licenses/by/3.0/).
